# Supplementary material for: A quantitative model used to compare within-host SARS-CoV-2, MERS-CoV, and SARS-CoV dynamics provides insights into the pathogenesis and treatment of SARS-CoV-2
Source: PLoS Biol. 2021 Mar 22;19(3):e3001128. doi: 10.1371/journal.pbio.3001128 (PMC7984623; doi:10.1371/journal.pbio.3001128)
Supplement: S1 Fig — (A–C) The estimated viral load for each individual patient (solid lines) along with the observed data (closed dots) are depicted using the best-fit parameter estimates. The dotted horizontal lines are the detection limits. Note that the detection limits for SARS-CoV-2 were 68 (Singapore, Korea), 15 (China), and 33.3 (Germany) copies/ml. The detection limits for MERS-CoV were 1,000 (Korea) and 15 (Saudi Arabia) copies/ml. The red closed dots at the detection limit represent the data points where the virus was detected but below the detection limit. The data underlying this figure are given in S4 Data. (DOCX) [file pbio.3001128.s011.docx]

**S1 Fig. Viral load trajectory for individual patients of SARS-CoV-2, MERS-CoV, and SARS-CoV.** **(A-C)** The estimated viral load for each individual patient (solid lines) along with the observed data (closed dots) are depicted using the best-fit parameter estimates. The dotted horizontal lines are the detection limits. Note that the detection limits for SARS-CoV-2 were 68 (Singapore, Korea), 15.3 (China) and 33.3 (Germany) copies/ml, respectively. The detection limits for MERS-CoV were 1,000 (Korea) and 15 (Saudi Arabia) copies/ml, respectively. The black closed dots represent the data points where the virus was detected but below the detection limit. The data underlying this Figure is given in S4 Data.
