# Supplementary figures and images for: A quantitative model used to compare within-host SARS-CoV-2, MERS-CoV, and SARS-CoV dynamics provides insights into the pathogenesis and treatment of SARS-CoV-2
Source: PLoS Biol. 2021 Mar 22;19(3):e3001128. doi: 10.1371/journal.pbio.3001128 (PMC7984623; doi:10.1371/journal.pbio.3001128)

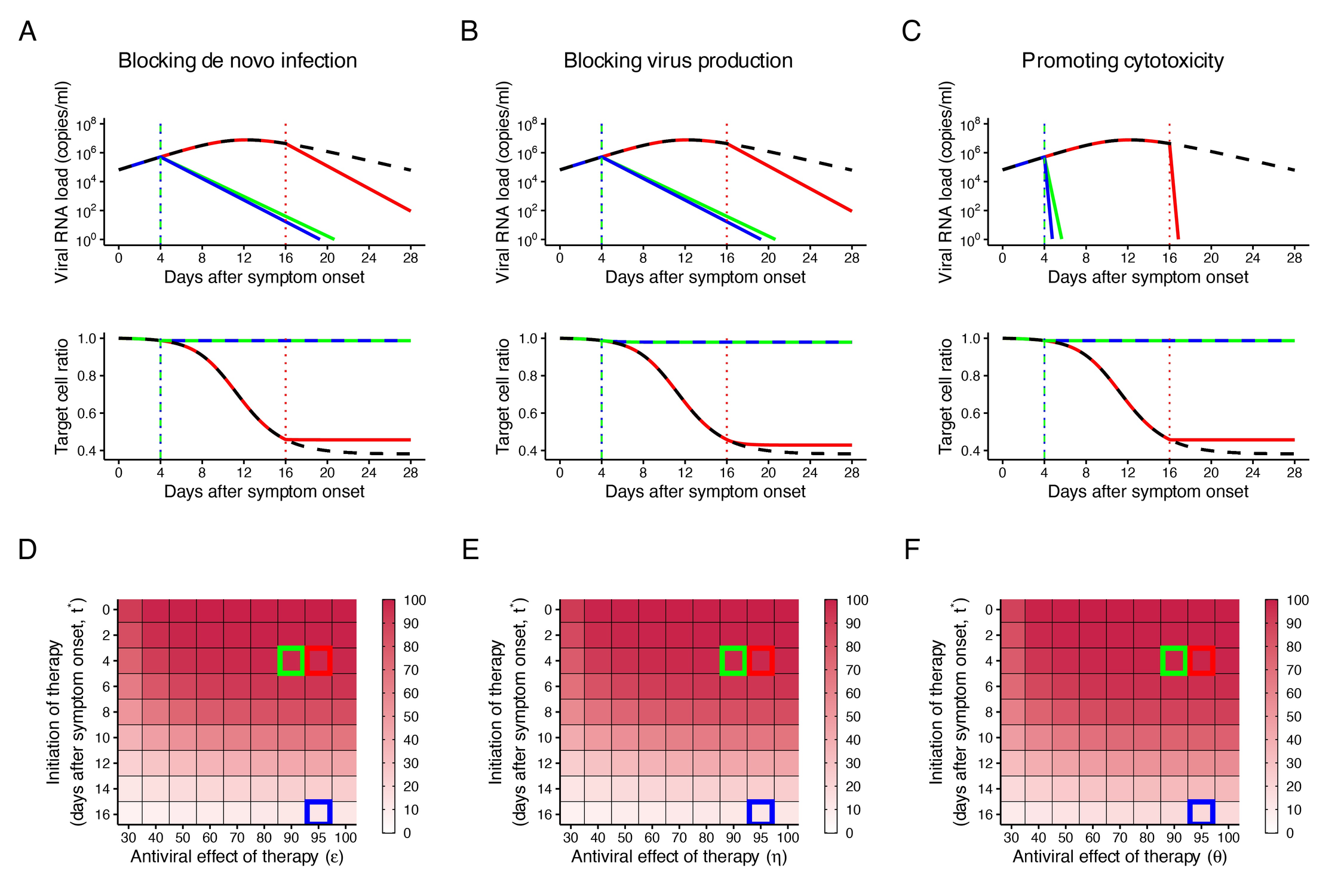

Supplement: S2 Fig — (A–C) Expected viral load and uninfected target cell proportion trajectories with and without treatment for the 3 different treatments. The black curves are without treatment. The blue curves are with treatment (efficacy is 95%) initiated at 16 days after symptom onset. The red and green curves are with treatment initiated at 4 days after symptom onset, but with different efficacy (95% and 90%). The dotted vertical lines correspond to the timing of treatment initiation. (D–F) The heatmap shows the reduction in the viral load AUC with treatment compared to without treatment. The timing of treatment initiation and treatment efficacy were varied. Darker colors indicate a larger reduction in the viral load AUC. The parameter setting used for the simulation in (A–C) is indicated by the same color in (D–F). The data underlying this figure are given in S5 Data. (TIF) [file pbio.3001128.s012.tif]

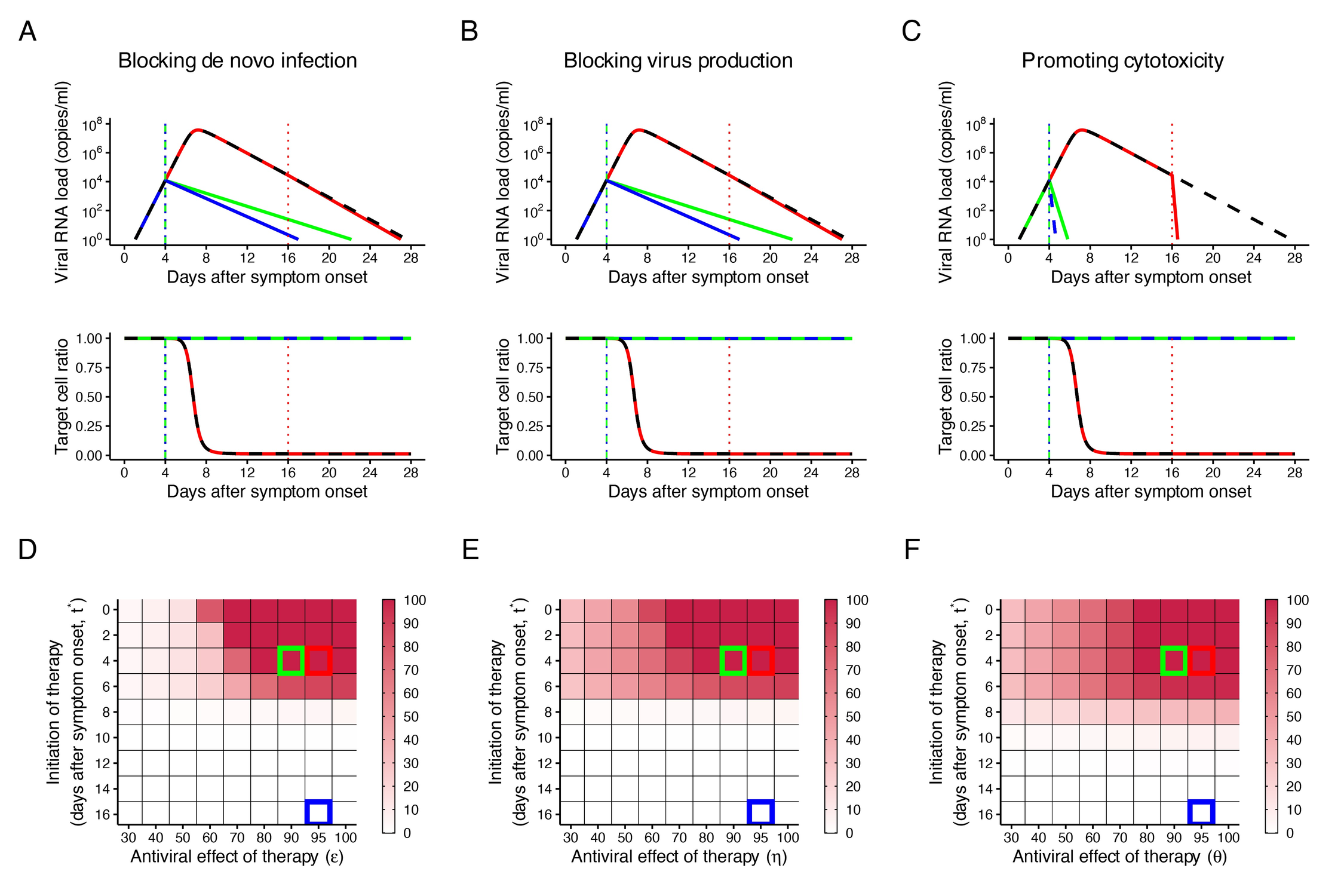

Supplement: S3 Fig — (A–C) Expected viral load and uninfected target cell proportion trajectories with and without treatment for the 3 different treatments. The black curves are without treatment. The blue curves are with treatment (efficacy is 95%) initiated at 16 days after symptom onset. The red and green curves are with treatment initiated at 4 days after symptom onset, but with different efficacy (95% and 90%). The dotted vertical lines correspond to the timing of treatment initiation. (D–F) The heatmap shows the reduction in the viral load AUC with treatment compared to without treatment. The timing of treatment initiation and treatment efficacy were varied. Darker colors indicate a larger reduction in the viral load AUC. The parameter setting used for the simulation in (A–C) is indicated by the same color in (D–F). The data underlying this figure are given in S6 Data. (TIF) [file pbio.3001128.s013.tif]

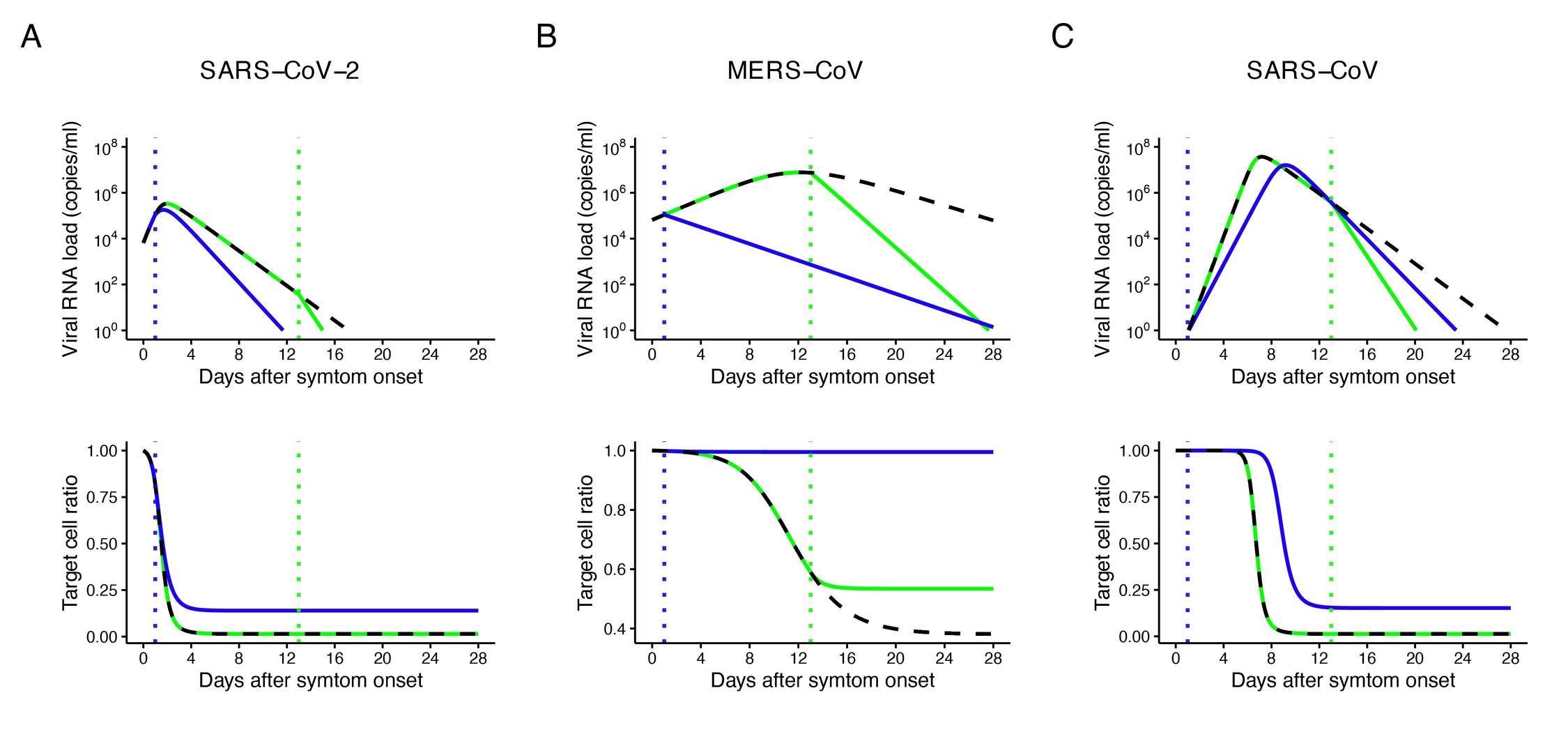

Supplement: S4 Fig — The dotted black curve corresponds to the viral load (top) and uninfected target cell proportion (bottom) trajectories without treatment promoting cytotoxicity for the 3 coronaviruses. The blue and green curves are with treatment with 50% efficacy initiated at 1 or 13 days after symptom onset. The data underlying this figure are given in S7 Data. (TIF) [file pbio.3001128.s014.tif]

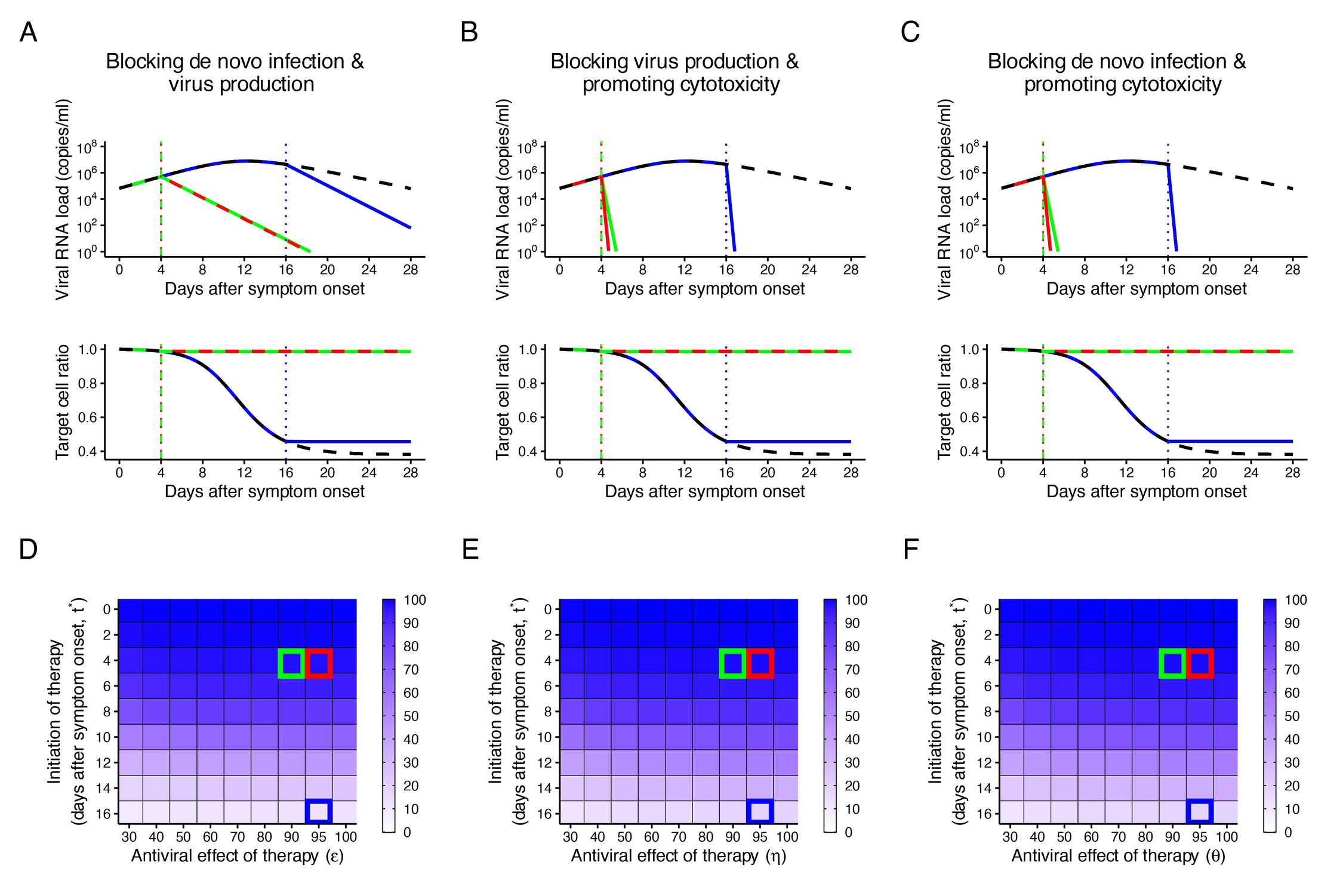

Supplement: S5 Fig — (A–C) Expected viral load and uninfected target cell proportion trajectories with and without treatment for the 3 combination therapies. We assumed the same efficacies and timing of treatment initiation for the 2 combined treatments. The black curves are without treatment. The blue curves are with treatment (efficacy is 95%) initiated at 16 days after symptom onset. The red and green curves are with treatment initiated at 4 days after symptom onset, but with different efficacy (95% and 90%). The dotted vertical lines correspond to the timing of treatment initiation. (D–F) The heatmap shows the reduction in the viral load AUC with treatment compared to without treatment. The timing of treatment initiation and treatment efficacy were varied. Darker colors indicate a larger reduction in the viral load AUC. The parameter setting used for the simulation in (A–C) is indicated by the same color in (D–F). The data underlying this figure are given in S8 Data. (TIF) [file pbio.3001128.s015.tif]

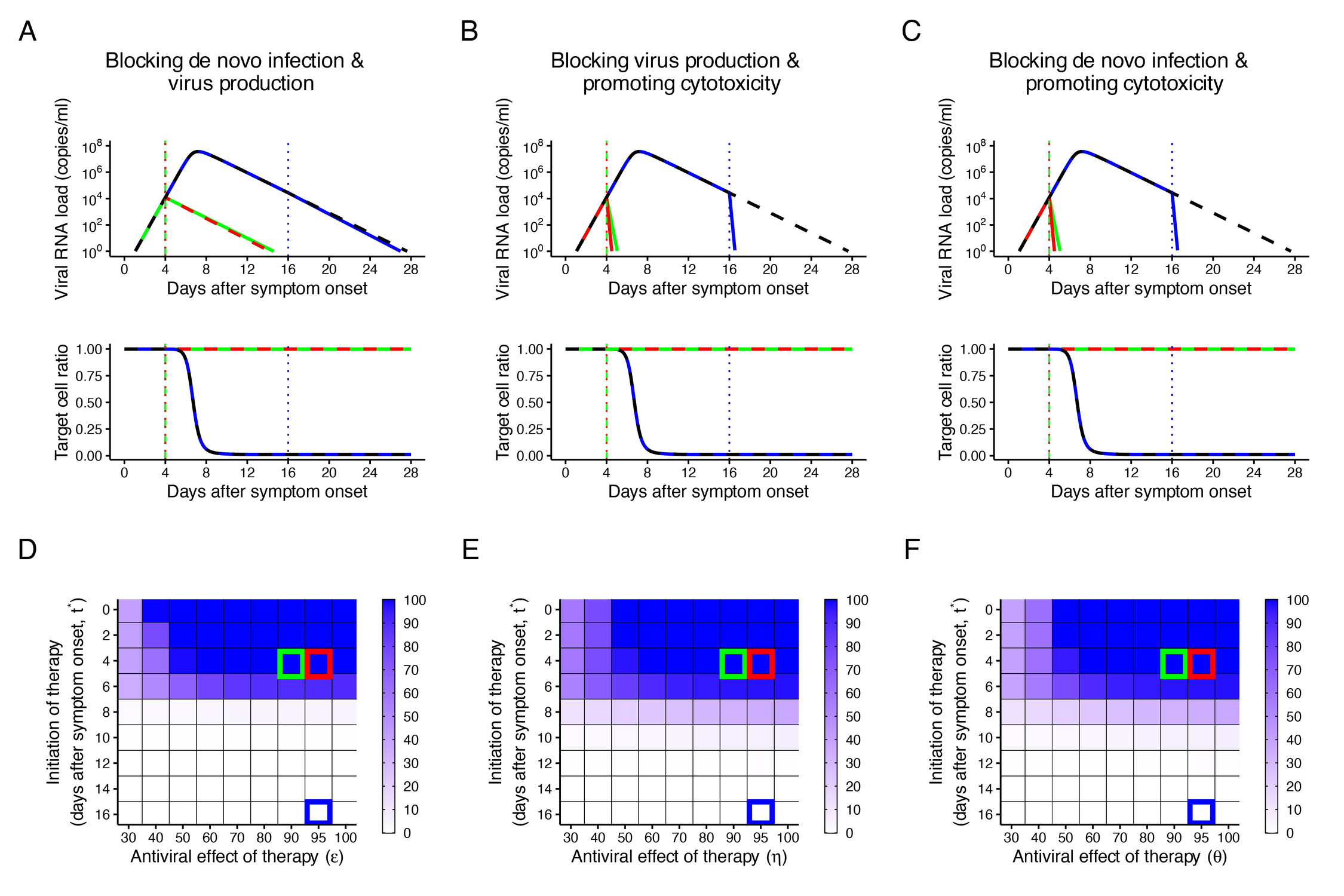

Supplement: S6 Fig — (A–C) Expected viral load and uninfected target cell proportion trajectories with and without treatment for the 3 combination therapies. We assumed the same efficacies and timing of treatment initiation for the 2 combined treatments. The black curves are without treatment. The blue curves are with treatment (efficacy is 95%) initiated at 16 days after symptom onset. The red and green curves are with treatment initiated at 4 days after symptom onset, but with different efficacy (95% and 90%). The dotted vertical lines correspond to the timing of treatment initiation. (D–F) The heatmap shows the reduction in the viral load AUC with treatment compared to without treatment. The timing of treatment initiation and treatment efficacy were varied. Darker colors indicate a larger reduction in the viral load AUC. The parameter setting used for the simulation in (A–C) is indicated by the same color in (D–F). The data underlying this figure are given in S9 Data. (TIF) [file pbio.3001128.s016.tif]

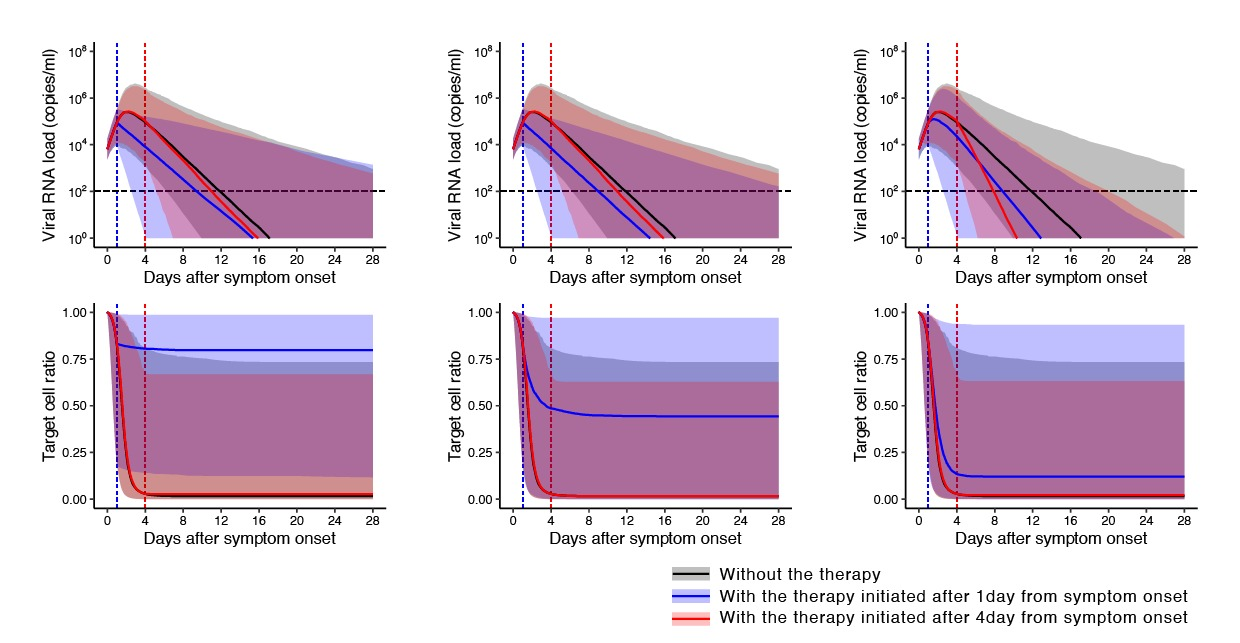

Supplement: S7 Fig — Expected viral load and uninfected target cell proportion trajectories with and without treatment for the 3 different treatments. The black curves are without treatment. The blue and red curves are with treatment (efficacy is 95%) initiated at 1 day and at 4 days after symptom onset, respectively. The shaded regions correspond to 95% predictive intervals. The data underlying this figure are given in S10 Data. (TIF) [file pbio.3001128.s017.tif]
