# Supplementary material for: A quantitative model used to compare within-host SARS-CoV-2, MERS-CoV, and SARS-CoV dynamics provides insights into the pathogenesis and treatment of SARS-CoV-2
Source: PLoS Biol. 2021 Mar 22;19(3):e3001128. doi: 10.1371/journal.pbio.3001128 (PMC7984623; doi:10.1371/journal.pbio.3001128)
Supplement: S1 Table — (DOCX) [file pbio.3001128.s018.docx]

**S1 Table | Summary of the data used in our analysis**

| **Virus type** | **Country** | **Number of cases included in the analysis** | **Specimens for measuring viral load** | **Papers** |
| --- | --- | --- | --- | --- |
| SARS-CoV-2 | Singapore | 12 | Nasopharyngeal swab | [[3](#_ENREF_3)] |
| SARS-CoV-2 | China | 8 | Nasal swab | [[4](#_ENREF_4)] |
| SARS-CoV-2 | Korea | 2 | Nasopharyngeal or oropharyngeal | [[1](#_ENREF_1)] |
| SARS-CoV-2 | Germany | 8 | Pharyngeal swab | [[2](#_ENREF_2)] |
| MERS-CoV | Korea | 7 | Sputum or Tracheal aspirate | [[5](#_ENREF_5)] |
| MERS-CoV | Saudi Arabia | 6 | Nasopharyngeal and oropharyngeal swab | [[6](#_ENREF_6)] |
| SARS-CoV | Hong Kong | 14 | Nasopharyngeal aspirate | [[7](#_ENREF_7)] |

**References**

1. Kim ES, Chin BS, Kang CK, Kim NJ, Kang YM, Choi JP, et al. Clinical Course and Outcomes of Patients with Severe Acute Respiratory Syndrome Coronavirus 2 Infection: a Preliminary Report of the First 28 Patients from the Korean Cohort Study on COVID-19. J Korean Med Sci. 2020;35(13):e142. Epub 2020/04/04. doi: 10.3346/jkms.2020.35.e142. PubMed PMID: 32242348; PubMed Central PMCID: PMCPMC7131901.

2. Wölfel R, Corman VM, Guggemos W, Seilmaier M, Zange S, Müller MA, et al. Virological assessment of hospitalized patients with COVID-2019. Nature. 2020;581(7809):465-9. Epub 2020/04/03. doi: 10.1038/s41586-020-2196-x. PubMed PMID: 32235945.

3. Young BE, Ong SWX, Kalimuddin S, Low JG, Tan SY, Loh J, et al. Epidemiologic Features and Clinical Course of Patients Infected With SARS-CoV-2 in Singapore. Jama. 2020;323(15):1488-94. Epub 2020/03/04. doi: 10.1001/jama.2020.3204. PubMed PMID: 32125362; PubMed Central PMCID: PMCPMC7054855 Sanofi and Roche. Dr Wang reported receiving grants from the Ministry of Health, Singapore. No other disclosures were reported.

4. Zou L, Ruan F, Huang M, Liang L, Huang H, Hong Z, et al. SARS-CoV-2 Viral Load in Upper Respiratory Specimens of Infected Patients. N Engl J Med. 2020;382(12):1177-9. Epub 2020/02/20. doi: 10.1056/NEJMc2001737. PubMed PMID: 32074444; PubMed Central PMCID: PMCPMC7121626.

5. Oh MD, Park WB, Choe PG, Choi SJ, Kim JI, Chae J, et al. Viral Load Kinetics of MERS Coronavirus Infection. N Engl J Med. 2016;375(13):1303-5. Epub 2016/09/30. doi: 10.1056/NEJMc1511695. PubMed PMID: 27682053.

6. Al-Abdely HM, Midgley CM, Alkhamis AM, Abedi GR, Lu X, Binder AM, et al. Middle East Respiratory Syndrome Coronavirus Infection Dynamics and Antibody Responses among Clinically Diverse Patients, Saudi Arabia. Emerg Infect Dis. 2019;25(4):753-66. Epub 2019/03/19. doi: 10.3201/eid2504.181595. PubMed PMID: 30882305; PubMed Central PMCID: PMCPMC6433025.

7. Peiris JS, Chu CM, Cheng VC, Chan KS, Hung IF, Poon LL, et al. Clinical progression and viral load in a community outbreak of coronavirus-associated SARS pneumonia: a prospective study. Lancet. 2003;361(9371):1767-72. Epub 2003/06/05. doi: 10.1016/s0140-6736(03)13412-5. PubMed PMID: 12781535; PubMed Central PMCID: PMCPMC7112410.
