# Supplementary material for: A quantitative model used to compare within-host SARS-CoV-2, MERS-CoV, and SARS-CoV dynamics provides insights into the pathogenesis and treatment of SARS-CoV-2
Source: PLoS Biol. 2021 Mar 22;19(3):e3001128. doi: 10.1371/journal.pbio.3001128 (PMC7984623; doi:10.1371/journal.pbio.3001128)
Supplement: S2 Table — (DOCX) [file pbio.3001128.s019.docx]

**S2 Table | Estimated parameters and initial values for each patient**

| **Country** | **Patient ID^#^** | $\boldsymbol{\gamma}$ | $\boldsymbol{\beta}$ | $\boldsymbol{\delta}$ | $\boldsymbol{V}\left( \boldsymbol{0} \right)$ | $\boldsymbol{L}$ | $\boldsymbol{R}_{\boldsymbol{0}}$ | $\boldsymbol{C}^{\boldsymbol{*}}$ | $\boldsymbol{T}_{\boldsymbol{p}}$ |
| --- | --- | --- | --- | --- | --- | --- | --- | --- | --- |
| **SAVS-CoV-2 patients** | | | | | | | | | |
| Singapore | 2 | $3.998$ | $6.64\times{10}^{-6}$ | $0.71$ | $6605$ | $1.41$ | $5.64$ | $0.82$ | $1.9$ |
| Singapore | 3 | $3.999$ | $1.26\times{10}^{-6}$ | $0.43$ | $6380$ | $2.35$ | $9.40$ | $0.89$ | $2.4$ |
| Singapore | 4 | $3.999$ | $5.81\times{10}^{-6}$ | $0.72$ | $6514$ | $1.39$ | $5.56$ | $0.82$ | $1.9$ |
| Singapore | 6 | $3.999$ | $3.08\times{10}^{-6}$ | $0.47$ | $6498$ | $2.11$ | $8.45$ | $0.88$ | $2.1$ |
| Singapore | 8 | $3.997$ | $1.96\times{10}^{-5}$ | $0.36$ | $6850$ | $2.75$ | $11.0$ | $0.91$ | $1.6$ |
| Singapore | 9 | $3.999$ | $1.56\times{10}^{-5}$ | $0.28$ | $6558$ | $3.56$ | $14.2$ | $0.93$ | $1.7$ |
| Singapore | 11 | $3.999$ | $8.46\times{10}^{-6}$ | $1.38$ | $6860$ | $0.72$ | $2.89$ | $0.65$ | $1.8$ |
| Singapore | 12 | $3.999$ | $7.77\times{10}^{-6}$ | $0.78$ | $6506$ | $1.28$ | $5.14$ | $0.81$ | $1.8$ |
| Singapore | 14 | $3.997$ | $3.52\times{10}^{-7}$ | $1.03$ | $5337$ | $0.97$ | $3.88$ | $0.74$ | $3.0$ |
| Singapore | 16 | $3.999$ | $4.88\times{10}^{-6}$ | $0.54$ | $6489$ | $1.85$ | $7.41$ | $0.87$ | $2.0$ |
| Singapore | 17 | $3.998$ | $1.32\times{10}^{-6}$ | $1.01$ | $5891$ | $0.99$ | $3.95$ | $0.75$ | $1.7$ |
| Singapore | 18 | $3.999$ | $5.80\times{10}^{-6}$ | $0.38$ | $6537$ | $2.61$ | $10.4$ | $0.90$ | $1.9$ |
| China | C | $4.002$ | $7.95\times{10}^{-6}$ | $1.29$ | $6596$ | $0.77$ | $3.10$ | $0.68$ | $1.9$ |
| China | D | $3.999$ | $1.69\times{10}^{-6}$ | $0.76$ | $6752$ | $1.31$ | $5.26$ | $0.81$ | $2.3$ |
| China | E | $3.999$ | $5.87\times{10}^{-6}$ | $0.84$ | $6517$ | $1.19$ | $4.75$ | $0.79$ | $1.9$ |
| China | H | $3.999$ | $1.05\times{10}^{-5}$ | $1.45$ | $7059$ | $0.69$ | $2.76$ | $0.64$ | $1.7$ |
| China | I | $3.999$ | $6.15\times{10}^{-7}$ | $0.45$ | $6251$ | $2.21$ | $8.85$ | $0.89$ | $2.6$ |
| China | L | $3.999$ | $1.73\times{10}^{-6}$ | $0.77$ | $6178$ | $1.29$ | $5.17$ | $0.81$ | $2.3$ |
| China | O | $3.999$ | $4.57\times{10}^{-5}$ | $1.90$ | $8541$ | $0.53$ | $2.10$ | $0.52$ | $0.9$ |
| China | P | $3.997$ | $1.02\times{10}^{-5}$ | $1.03$ | $6808$ | $0.97$ | $3.88$ | $0.74$ | $1.7$ |
| Germany | 1 | $3.999$ | $7.28\times{10}^{-6}$ | $0.98$ | $6610$ | $1.02$ | $4.08$ | $0.75$ | $1.9$ |
| Germany | 2 | $3.999$ | $2.88\times{10}^{-6}$ | $1.51$ | $6262$ | $0.66$ | $2.64$ | $0.62$ | $2.4$ |
| Germany | 3 | $3.996$ | $1.11\times{10}^{-5}$ | $1.32$ | $5894$ | $0.76$ | $3.02$ | $0.67$ | $1.8$ |
| Germany | 4 | $4.000$ | $6.65\times{10}^{-6}$ | $1.60$ | $6678$ | $0.63$ | $2.50$ | $0.60$ | $2.0$ |
| Germany | 7 | $3.999$ | $3.08\times{10}^{-6}$ | $1.11$ | $6259$ | $0.90$ | $3.02$ | $0.72$ | $2.2$ |
| Germany | 8 | $3.999$ | $3.29\times{10}^{-6}$ | $1.08$ | $6375$ | $0.93$ | $2.50$ | $0.73$ | $2.2$ |
| Germany | 10 | $3.999$ | $2.67\times{10}^{-6}$ | $0.61$ | $6268$ | $1.64$ | $3.62$ | $0.85$ | $2.2$ |
| Germany | 14 | $3.996$ | $8.91\times{10}^{-6}$ | $1.61$ | $6350$ | $0.62$ | $3.71$ | $0.60$ | $1.9$ |
| Korea | 13 | $3.998$ | $9.67\times{10}^{-6}$ | $1.15$ | $7674$ | $0.87$ | $6.56$ | $0.71$ | $1.7$ |
| Korea | 15 | $3.998$ | $1.44\times{10}^{-5}$ | $1.16$ | $5802$ | $0.86$ | $2.47$ | $0.71$ | $2.5$ |
| **MERS-CoV patients** | | | | | | | | | |
| Korea | 1 | $1.450$ | $2.29\times{10}^{-9}$ | $0.57$ | $67368$ | $1.76$ | $2.56$ | $0.61$ | $10.9$ |
| Korea | 2 | $1.450$ | $1.85\times{10}^{-8}$ | $0.69$ | $70456$ | $1.45$ | $2.10$ | $0.52$ | $9.2$ |
| Korea | 3 | $1.450$ | $5.30\times{10}^{-9}$ | $0.69$ | $64614$ | $1.44$ | $2.09$ | $0.52$ | $11.0$ |
| Korea | 4 | $1.450$ | $1.39\times{10}^{-8}$ | $0.65$ | $66189$ | $1.54$ | $2.23$ | $0.55$ | $9.4$ |
| Korea | 5 | $1.450$ | $7.75\times{10}^{-9}$ | $0.69$ | $65218$ | $1.45$ | $2.10$ | $0.52$ | $10.5$ |
| Korea | 10 | $1.451$ | $1.85\times{10}^{-7}$ | $0.86$ | $83978$ | $1.16$ | $1.69$ | $0.41$ | $6.2$ |
| Saudi Arabia | 2 | $1.450$ | $1.41\times{10}^{-8}$ | $1.97$ | $79805$ | $0.51$ | $0.73$ | $-0.36$ | $0$ |
| Saudi Arabia | 23 | $1.449$ | $1.23\times{10}^{-8}$ | $1.38$ | $55807$ | $0.72$ | $1.05$ | $0.05$ | $30.1$ |
| Saudi Arabia | 24 | $1.450$ | $1.39\times{10}^{-8}$ | $2.62$ | $50058$ | $0.38$ | $0.55$ | $-0.80$ | $0$ |
| Saudi Arabia | 25 | $1.450$ | $1.39\times{10}^{-8}$ | $2.25$ | $55811$ | $0.44$ | $0.64$ | $-0.55$ | $0$ |
| Saudi Arabia | 26 | $1.450$ | $1.40\times{10}^{-8}$ | $2.12$ | $63222$ | $0.47$ | $0.68$ | $-0.46$ | $0$ |
| Saudi Arabia | 29 | $1.450$ | $1.40\times{10}^{-8}$ | $2.06$ | $68319$ | $0.49$ | $0.70$ | $-0.42$ | $0$ |
| Saudi Arabia | 39 | $1.450$ | $1.40\times{10}^{-8}$ | $2.42$ | $80914$ | $0.41$ | $0.60$ | $-0.67$ | $0$ |
| **SAVS-CoV patients** | | | | | | | | | |
| Hong Kong | A | $4.129$ | $1.81\times{10}^{-8}$ | $0.91$ | $0.03$ | $1.10$ | $4.55$ | $0.78$ | $7.5$ |
| Hong Kong | B | $4.129$ | $2.42\times{10}^{-8}$ | $0.82$ | $0.03$ | $1.22$ | $5.04$ | $0.80$ | $7.3$ |
| Hong Kong | C | $4.134$ | $1.17\times{10}^{-7}$ | $0.82$ | $0.04$ | $1.22$ | $5.05$ | $0.80$ | $6.7$ |
| Hong Kong | D | $4.131$ | $3.43\times{10}^{-8}$ | $1.52$ | $0.03$ | $0.66$ | $2.71$ | $0.63$ | $8.7$ |
| Hong Kong | E | $4.130$ | $4.55\times{10}^{-8}$ | $1.41$ | $0.03$ | $0.71$ | $2.94$ | $0.66$ | $8.3$ |
| Hong Kong | F | $4.132$ | $2.32\times{10}^{-8}$ | $0.75$ | $0.03$ | $1.34$ | $5.52$ | $0.82$ | $7.1$ |
| Hong Kong | G | $4.133$ | $9.44\times{10}^{-8}$ | $0.90$ | $0.09$ | $1.11$ | $4.05$ | $0.78$ | $6.9$ |
| Hong Kong | H | $4.131$ | $4.23\times{10}^{-8}$ | $0.92$ | $0.03$ | $1.09$ | $3.97$ | $0.78$ | $7.2$ |
| Hong Kong | I | $4.132$ | $1.00\times{10}^{-7}$ | $1.72$ | $0.03$ | $0.58$ | $2.49$ | $0.58$ | $8.9$ |
| Hong Kong | J | $4.132$ | $2.14\times{10}^{-8}$ | $0.57$ | $0.03$ | $1.76$ | $7.27$ | $0.86$ | $6.9$ |
| Hong Kong | K | $4.133$ | $2.36\times{10}^{-8}$ | $0.52$ | $0.03$ | $1.93$ | $7.98$ | $0.87$ | $6.8$ |
| Hong Kong | L | $4.130$ | $6.53\times{10}^{-8}$ | $1.13$ | $0.03$ | $0.89$ | $3.66$ | $0.73$ | $7.5$ |
| Hong Kong | M | $4.133$ | $6.30\times{10}^{-8}$ | $0.89$ | $0.03$ | $1.12$ | $4.67$ | $0.79$ | $7.0$ |
| Hong Kong | N | $4.133$ | $3.35\times{10}^{-8}$ | $0.57$ | $0.04$ | $1.76$ | $7.29$ | $0.86$ | $6.7$ |

^#^ The patients’ IDs correspond to those in the original reports.
