# Supplementary material for: A quantitative model used to compare within-host SARS-CoV-2, MERS-CoV, and SARS-CoV dynamics provides insights into the pathogenesis and treatment of SARS-CoV-2
Source: PLoS Biol. 2021 Mar 22;19(3):e3001128. doi: 10.1371/journal.pbio.3001128 (PMC7984623; doi:10.1371/journal.pbio.3001128)
Supplement: S1 Text — (DOCX) [file pbio.3001128.s020.docx]

**S1 Text: Study data**

We searched for longitudinal viral load data of SARS-CoV-2, SARS-CoV, and MERS-CoV patients. We set five inclusion criteria in searching the relevant datasets: 1. viral load was reported (if cycle thresholds were reported instead of viral load, they were transformed to viral load using the conversion formula[1]: $\log_{10} \left( Viral load [copies/ml] \right)=-0.32*Ct values [cycles]+14.11$), 2. viral load was measured from respiratory specimens for consistency, 3. viral load along with the time since symptom onset was reported, 4. patients with more than three data points for parameter fitting, 5. patients not under antiviral treatment, because antiviral therapy can directly influence the viral dynamics and the antiviral effect cannot be modeled properly without detailed information such as the treatment schedule, mode of action, dose, and drug efficacy. For all the studies from which we extracted data, ethics approval was obtained from the ethics committee at each institution. Written informed consent was obtained from the cases or their next of kin in the original studies. Using these criteria we identified 57 patients from 7 papers. We summarized the data in **S1 Table**. Followings are more detail of the data sets.

SARS-CoV-2:

The viral load data from hospitalized COVID-19 patients in four previously published papers were used [[1-4](#_ENREF_1)]. All patients used in our analysis presented symptoms before or after hospitalization. For consistency, the viral load data from upper respiratory specimens were used in the analysis. Eight cases reported from China and one case reported from Germany were excluded because their viral loads were above the detection limit only twice or less [[2](#_ENREF_2), [4](#_ENREF_4)]. Five patients from Singapore who received lopinavir-ritonavir treatment and one patient with less than two data points were excluded [[3](#_ENREF_3)].

MERS-CoV:

The viral load data from two previously published papers from hospitalized MERS patients were used [[5](#_ENREF_5), [6](#_ENREF_6)]. We excluded Patients 9 and 8 from Korea and Saudi Arabia as they had less than two data points.

SARS-CoV:

The viral load data from hospitalized SARS-CoV patients in one previously published paper were used [[7](#_ENREF_7)].

**References**

1. Kim ES, Chin BS, Kang CK, Kim NJ, Kang YM, Choi JP, et al. Clinical Course and Outcomes of Patients with Severe Acute Respiratory Syndrome Coronavirus 2 Infection: a Preliminary Report of the First 28 Patients from the Korean Cohort Study on COVID-19. J Korean Med Sci. 2020;35(13):e142. Epub 2020/04/04. doi: 10.3346/jkms.2020.35.e142. PubMed PMID: 32242348; PubMed Central PMCID: PMCPMC7131901.

2. Wölfel R, Corman VM, Guggemos W, Seilmaier M, Zange S, Müller MA, et al. Virological assessment of hospitalized patients with COVID-2019. Nature. 2020;581(7809):465-9. Epub 2020/04/03. doi: 10.1038/s41586-020-2196-x. PubMed PMID: 32235945.

3. Young BE, Ong SWX, Kalimuddin S, Low JG, Tan SY, Loh J, et al. Epidemiologic Features and Clinical Course of Patients Infected With SARS-CoV-2 in Singapore. Jama. 2020;323(15):1488-94. Epub 2020/03/04. doi: 10.1001/jama.2020.3204. PubMed PMID: 32125362; PubMed Central PMCID: PMCPMC7054855 Sanofi and Roche. Dr Wang reported receiving grants from the Ministry of Health, Singapore. No other disclosures were reported.

4. Zou L, Ruan F, Huang M, Liang L, Huang H, Hong Z, et al. SARS-CoV-2 Viral Load in Upper Respiratory Specimens of Infected Patients. N Engl J Med. 2020;382(12):1177-9. Epub 2020/02/20. doi: 10.1056/NEJMc2001737. PubMed PMID: 32074444; PubMed Central PMCID: PMCPMC7121626.

5. Oh MD, Park WB, Choe PG, Choi SJ, Kim JI, Chae J, et al. Viral Load Kinetics of MERS Coronavirus Infection. N Engl J Med. 2016;375(13):1303-5. Epub 2016/09/30. doi: 10.1056/NEJMc1511695. PubMed PMID: 27682053.

6. Al-Abdely HM, Midgley CM, Alkhamis AM, Abedi GR, Lu X, Binder AM, et al. Middle East Respiratory Syndrome Coronavirus Infection Dynamics and Antibody Responses among Clinically Diverse Patients, Saudi Arabia. Emerg Infect Dis. 2019;25(4):753-66. Epub 2019/03/19. doi: 10.3201/eid2504.181595. PubMed PMID: 30882305; PubMed Central PMCID: PMCPMC6433025.

7. Peiris JS, Chu CM, Cheng VC, Chan KS, Hung IF, Poon LL, et al. Clinical progression and viral load in a community outbreak of coronavirus-associated SARS pneumonia: a prospective study. Lancet. 2003;361(9371):1767-72. Epub 2003/06/05. doi: 10.1016/s0140-6736(03)13412-5. PubMed PMID: 12781535; PubMed Central PMCID: PMCPMC7112410.
