# Supplementary material for: A quantitative model used to compare within-host SARS-CoV-2, MERS-CoV, and SARS-CoV dynamics provides insights into the pathogenesis and treatment of SARS-CoV-2
Source: PLoS Biol. 2021 Mar 22;19(3):e3001128. doi: 10.1371/journal.pbio.3001128 (PMC7984623; doi:10.1371/journal.pbio.3001128)
Supplement: Attachment — Submitted filename: PBP_submit.pdf [file pbio.3001128.s021.pdf]

<u>Response to reviewer comments</u>

Kwang Su Kim, Keisuke Ejima, Shoya Iwanami, Yasuhisa Fujita, Hirofumi Ohashi, Yoshiki Koizumi, Yusuke Asai, Shinji Nakaoka, Koichi Watashi, Kazuyuki Aihara, Robin N. Thompson, Ruian Ke, Alan S. Perelson and Shingo Iwami

December 8, 2020

We would like to thank the reviewers for their thorough reading of the manuscript and their excellent comments and suggestions. In this letter, we present the reviewers' comments in black and provide our responses in red. All the changes made in response to the reviewers' comments both in PBIOLOGY-D-20-01314R1 and PNAS MS# 2020-04882 (see later) have been addressed in the revised version of the manuscript. We believe that the manuscript has been considerably improved by these changes. All coauthors agreed with these changes.

Editor Remarks to Author:

I should make it clear that reviewer #1 informed us that s/he had seen this manuscript elsewhere, but because you had not addressed any of the concerns raised, they simply provided their previous review. We also recruited a third reviewer; however, they also realized that they had seen the paper at another journal, and because none of their concerns had been addressed, they declined to submit a review.

Reply to Editor Remarks:

After first viral load data of COVID-19 patients published in [1] Feb19, we immediately analyzed them, and submitted the results to PNAS as "PNAS MS# 2020-04882". Our paper was sent to external review but was rejected mainly because of the limited size of the datasets. At that time, it was difficult to find additional datasets, but now several more datasets are available to address all the comments from the reviewers in PBIOLOGY-D-20-01314R1 as well as PNAS MS# 2020-04882. We have significantly improved this paper with the additional data and analyses which, we believe, satisfy all the prior reviewers. Please find our appeal and reconsider our revised manuscript.

You'll see that both of the reviewers raise significant concerns about the manuscript, but the Academic Editor particularly focused on the following reviewer comments when advising us not to consider your manuscript further:

1. "As a result, the major conclusion that SARS-CoV-2 has more severe infection dynamics that mild MERS does not seem very robust or to add more insight than just looking at viral loads."

Reply to Editor comment 1:

In our current analysis, the viral load data from six published papers (only a single paper was used in our previous version) of hospitalized COVID-19 patients were used [1-6]. We also added MERS-CoV data from Saudi Arabia [7]. For consistency, the viral load data from upper respiratory specimens were used in the analysis except for the MERS-CoV data from Korea which is from sputum or tracheal aspirate. We avoided discussing the "severity" of SARS-CoV-2 since the data we have analyzed with regard to SARS-CoV and SARS-CoV-2 is not annotated with patients' severity. Here we focused on the quantitative comparison

of SARS-CoV-2, MERS-CoV and SARS-CoV infection dynamics. In this revised manuscript, we have now computed the time of the viral load peak relative to the time of symptom onset. We found the peak comes earlier for SARS-CoV-2 cases than for SARS-CoV or MERS. Combined with the finding of a larger reproduction number at symptom onset, R_{S0}, our findings suggest the difficulty of using antiviral therapy to control SARS-CoV-2 infection compared with the other two coronaviruses.

2.“Since the parameter space seems very flat for most of the models, comparing whether 'virus' (ie: SARS1, SARS2, MERS) is a significant factor in fitting a non-linear mixed effects model to the entire data set seems important. (they also pool both mild and severe MERS in the mixed effects - so they presumably did not address the central conclusion of the manuscript using this approach).”

Reply to Editor comment 2:

In this new manuscript, the difference among the three coronaviruses became clearer. In particular, we find the reproduction number at symptom onset (R_{S0}) and the time from symptom onset to the viral load peak differentiates the dynamics of SARS-CoV-2 from that of SARS-CoV and MERS. As we did not have data on the severity of COVID-19 in SARS-CoV-2 and SARS-CoV patients, the mild and severe cases of MERS were combined in this revised manuscript.

3. “With the one drug class that does show an effect with late initiation, I found the prediction of the viral load declining faster with late initiation (and even crossing over) than with early initiation difficult to understand (Fig. 3C). The authors do not offer an explanation of this behavior, which they should be able to using their model. Thus, I find the inferences of the effects of different drug classes and timing of treatment initiation not satisfactory.”

Reply to Editor comment 3:

In our revised work reported on here, we found an interesting property for therapies that increase the death rate of infected cells. If drugs promoting cytotoxicity are initiated after the peak viral load (i.e., late initiation), then the drugs effectively enhance viral decay (since the cells producing virus are more rapidly killed) and reduce the cumulative viral load (i.e., AUC). While, if the drugs were initiated before the viral load peak (i.e., early initiation), then the drugs

delayed the time of viral load peak rather than enhancing the rate of decay of the viral load, and therefore had relatively mild effects on AUC reduction. Because drugs promoting cytotoxicity cannot block ongoing *de novo* infection, the antiviral effects depend on target cell availability. In other words, because a large fraction of target cells remain at the early phase of infection, ongoing *de novo* infection is not blocked by drugs enhancing clearance of infected cells and infection continues albeit at slower speed. The revised manuscript explains these effects in detail.

Data from 66 individuals are now analyzed yielding more robust results:

We used 39 cases in which viral loads were measured using nasal swabs (China [1]), pharyngeal swabs (Germany [5]), nasopharyngeal swabs (Singapore [6], France [4], USA [2]) and nasopharyngeal and oropharyngeal swabs (Korea [3]) from SARS-CoV-2 infected patients, 13 cases of viral load from sputum or tracheal aspirate (Korea [8]) and nasopharyngeal and oropharyngeal swabs (Saudi Arabia [7]) from MERS-CoV infected patients, and 14 cases of viral load from nasopharyngeal aspirate (Hong Kong [9]) from SARS-CoV infected patients, respectively. The data are summarized in **Table S1**.

Additional quantitative analysis for combination therapies:

Effective combinations of anti-SARS-CoV-2 therapies might enhance the antiviral effects and/or reduce the individual drug dose and/or the side effects, all of which are highly desirable. This is expected when the modes of action are not the same between drugs. To evaluate whether combination therapies provide better outcomes than monotherapies, we have now expanded our paper to include quantitative analysis of combination therapies for SARS-CoV-2 as well as MERS-CoV and SARS-CoV.

Major changes in revision:

We added the following results in the Text and Supporting Information during revision:

1. (iv) Combination therapy in Results and Discussion and Methods
2. Figure 3
3. Figure S1
4. Figure S2

5. Figure S3
6. Figure S4
7. Figure S5
8. Figure S6

Reviewer #1 in PBIOLOGY-D-20-01314R1:
Reviewer #1 in PNAS MS# 2020-04882 as well:
Covid-19 represents a major human health challenge and timely and informative assessment of viral dynamics could accelerate the development of therapeutics. This manuscript addresses that question using published data on SARS, SARS-CoV-2, and MERS. It comes to a number of conclusions about differences in viral dynamics between infections as well as the potential impact of interventions. The major weaknesses of the work are the poor data input, and the very poor fit of the model to the data.

Reply to Reviewer summary:

We are glad that the reviewer recognized the importance of the study's purpose. We agree the sample size of the data was small in our previous analysis. In this revised manuscript, longitudinal viral load data from hospitalized COVID-19 patients described in six published papers (it was only a single paper previously) were used [1-6]. We also included additional MERS-CoV data from Saudi Arabia [7]. In total, we collected and analyzed the longitudinal viral load data from 39 SARS-CoV-2 cases, 13 MERS-CoV cases, and 14 SARS-CoV cases. The data are summarized in **Table S1**.

【Major issues】

1. A major conclusion of the data relates to comparing different viruses, and in the context of SARS-CoV-2 the comparison with mild / severe MERS is the major one discussed. However, there are major differences in the data sets that make this comparison difficult.

   a. The detection limits for SARS-CoV-2 is 15 copies versus 1000 copies for the other viruses. Six of 14 SARS-CoV-2 patients have no values >1000, and another 5 have only 1 value >1000. This leaves 3 patients with > 1 value that would have been detected in the other assays.

Reply to major comment 1-a:

As the reviewer noted, the viral load was not measured using the same lower threshold in different studies. However, we do not think this limits the comparison between the different viruses. The utility of mathematical model is

that by fitting all of data simultaneously using a population approach in the context of a mixed-effects model we can use the model to estimate the viral load even if some data is missing or under the detection limit. Thus, even though the data above the detection limited is scarce for some cases, we can robustly estimate the parameters for those cases. Further, the data under detection limits were treated properly using a commonly used approach in this field for handling censored data (see **Method section**).

b. The data for mild MERS-CoV has only 4 datapoints that exceed the detection limit (two of which are in the same patient). Put alternatively, the data includes 5/8 patients for whom virus never exceeded the detection limit (and the model predicts a rising viral load after cessation of collection in these patients - 2 -4 weeks after symptoms). As a result, the major conclusion that SARS-CoV-2 has more severe infection dynamics that mild MERS does not seem very robust or to add more insight than just looking at viral loads.

Reply to major comment 1-b:

We agree that including patients without data above detection limit does not make sense. In this version, only data from patients with more than three data points above the detection limit were included in the analysis. Further, the data from patients treated with antivirals were excluded (see **Study data** in **Supplementary Material**). We also analyzed the data of mild and severe cases of MERS together, because patients' severity was not reported for the other two coronaviruses.

2. The authors say that they use a mixed effects model, but then do not state whether 'virus' as a fixed effect is significant. They instead appear to plot central estimates from bootstrapping and compare these using a t test. Since the parameter space seems very flat for most of the models, comparing whether 'virus' (ie: SARS1, SARS2, MERS) is a significant factor in fitting a non-linear mixed effects model to the entire dataset seems important. (they also pool both mild and severe MERS in the mixed effects - so they presumably did not address the central conclusion of the manuscript using this approach).

Reply to major comment 2:

In the revised mixed effect model, the different viruses were used as fixed effects (categorical variable). We then tested each of the model parameters to determine if they covaried with the virus type. There was statistical support (i.e., significant difference) for the parameters γ, β and $V(0)$, to covary with the virus type (see **Table 1**).

3. Table S1 shows a variety of parameter estimates for the different infections. A number of the columns seem redundant (L = 1/ delta, C = 1/R0). Parameters estimates for beta in SARS-CoV-2 vary by >10e4, and estimates for V0 vary by 10e10.

Reply to major comment 3:

We agree that the mean length of virus production of an infected cell ($L = 1/\delta$) and the death rate of infected cells δ are mathematically identical (as well as R_{S0} and C^*). However, the biological meanings of these indices are important in this study. Thus, we choose to retain them in **Table S1**. Also, note that the values of our estimated parameters have been updated from those in previous analysis and for example the estimates of beta for SARS-CoV-2 now cluster around a few times 10e-6 with a few outliers and the estimates of V(0) for SARS-CoV-2 vary by less than 1e4. Because t=0 is the time of symptom onset, we do not find it surprising that V(0) estimates vary; recall some individuals with high viral loads never show symptoms.

4. The model is essentially a 'target cell limited' one. However, it seems implicitly assumed that the number of target cells is the same for everyone (and thus viral load can only vary with viral parameters). It also assumes no immune response (even though many of the peaks are predicted to occur >10 days from symptoms (which are 5-7 days from infection)). The assumption that decline in viral levels occurs due to target cell destruction limits analysis of drug effects. In addition, although the basal turnover of 'target' cells is assumed to be low, the homeostatic replacement rate might be quite high (and would affect interpretation).

Reply to major comment 4:

We agree that our simple mathematical model has limitations. However, target cell limited models have proved very valuable in understanding infection dynamics and therapy for HIV [10, 11], HCV [12], influenza [13], West Nile virus [14] Zika virus [15] and for SARS-CoV-2 [16-18]. Although the model does not explicitly describe immune responses, the effects of immune responses are implicitly included in model parameters such as the infection rate, which can be influenced by innate responses, and the death rate of infected cells, which can be influenced by adaptive immune responses. Because of the simplicity of the model, the model parameters can be estimated and compared among the various coronaviruses. These points are now incorporated into the text at the beginning of the methods section. Next steps will be to explicitly consider immune responses in models after they become better studied. For influenza, which also infects epithelial cells in the respiratory system, a model that included logistic proliferation of target cells was tested and rejected because the estimated growth rate was not significantly different than zero [13]. Also, observations in the human trachea after influenza infection suggest epithelial cell replenishment mainly occurs after the infection is cleared. Lastly, target cell limitation does not imply that all epithelial cells need to die to limit infection. For the beta coronaviruses, only a small fraction of all epithelial cells carry the ACE2 receptor and the required protease enzymes need for viral entry and thus are target cells for these viruses.

In principle, when relevant data becomes available for the three coronaviruses we study here, the effects the reviewer mentions could be included in an extended version of our model. However, without experimentally or clinically validated datasets, building a complicated model may end up including nonlinear terms that are unvalidated and potentially far from reality. We also would face the difficulty of identifiability of parameter as we discussed in our recent paper (S Iwanami et al., 2020 in PLOS Biology). Further, in recent reports, different and more complicated mathematical models reached similar conclusions especially about the timing of drug initiation needed to reduce SARS-CoV-2 viral load [16, 19], suggesting robustness of our analyses here.

We added material to clarify that there are the limitations of our mathematical model but that meaningful insights into COVID-19 therapies can still be obtained (See Lines 329-338on Page 16).

5. The conclusions about drug effects are very dependent on assumptions of viral dynamics. For example, there appear to be only 2 SARS-CoV-2 patients

where there is a meaningful rise in virus (others have a one-off high value). Yet conclusions about the relative impact of different drugs is dependent on the assumption of viral growth after presentation. The assumption of target cell limitation also strongly affects the interpretation of potential drug effects.

Reply to major comment 5:

In our revised manuscript, we now analyze longitudinal viral load data from six published studies involving hospitalized COVID-19 patients [1-6]. We also added MERS-CoV data from Saudi Arabia [7]. Using the simple (but reasonable at least for these coronavirus infections) mathematical model in combination with these published viral load data collected from the same specimen, we now obtained reasonable and robust parameter estimations for SARS-CoV-2, MERS-CoV, and SARS-CoV infection.

6. This is an important question and the authors apply a rational approach to analysis. It is a pity that limitations in data fitting make it difficult to draw any meaningful conclusions.

Reply to major comment 6:

Thank you for the positive comment on our mathematical model-based quantitative data analysis for SARS-CoV-2, MERS-CoV, and SARS-CoV infection. We agree available data were limited when we first submitted our manuscript. But now we use several additional data sets to address all the reviewer's comments.

Reviewer #2 in PBIOLOGY-D-20-01314R1:

In this article, the authors apply standard models of viral dynamics to data on SARS-CoV-2, MERS-CoV, and SARS-CoV viral load from infected patients and draw inferences on the relative severity of the infections. They then use the parameters estimated for SARS-CoV-2 and make predictions of the potency of different types of interventions for SARS-CoV-2 infection. Their main conclusions are: 1) SARS-CoV-2 infection is more severe than SARS-CoV infection and comparable to severe MERS-CoV infection; 2) Different classes of drugs would elicit different responses in curtailing overall viral load; 3) Timing of treatment initiation can be critical to ensuring successful treatment. The data analysis is done using mixed-effects modeling in Monolix, which is well equipped to handle sparse datasets and yield population level parameters. Previously published data on all three infections is used. The paper is well written.

Reply to Reviewer summary:

Thank you for your positive comments and giving us constructive comments to improve the quality of our research. In this revised version, we have added more data from papers published after the initial submission. Further, we restricted the analysis for data from patients with more than three data points above the detection limit to reliably estimate parameters for each individual. We have updated the manuscript to clarify the data, analytical protocol, and interpretation of the results.

I found the data analysis well done and the inferences there reliable (conclusion 1). I am not convinced, however, about the predictions made on the outcomes of treatment and optimal timings (conclusions 2 and 3). On the data analysis, too, more care is necessary in placing the results in the right context. My specific comments are below.

【Comments】

1. I wish to draw the attention of the authors to the following preprint: https://www.medrxiv.org/content/10.1101/2020.04.04.20047886v1. Here the authors apply viral dynamics models to analyze patient data on SARS-CoV-2 viral load measurements following treatment with many different drugs. Estimates of R0 are obtained and the effect of the timing of therapy initiation

is predicted. Median R0 was ~13 (range 2.3 - 46.7), which is at odds with the estimates obtained in the present study (much lower). Further, late treatment initiation was found to result in worse outcomes than early treatments, similar to what is presently claimed, but based on a direct estimate of viral dynamics parameters from patient data. Also, the present study claims that with one class of drugs late treatment initiation may be beneficial, which may be difficult to justify. The authors must comment on how their findings compare with the results in the above preprint.

Reply to major comment 1:

We found the suggested paper is now published (https://ascpt.onlinelibrary.wiley.com/doi/10.1002/psp4.12543). In the published version the estimate of R0 is 8.6 (with a 95% CI of 1.9-17.6), which is lower than in the preprint. Further, they assumed a constant 5 day incubation period. Given that incubation period varies between patients, it is not appropriate to use the fixed value. Further, the initial viral load at the time of infection was also fixed (i.e., the same for all the patients), which is biologically unmeasurable. In our study, we used the time from symptom onset as the time scale and the initial viral load at symptom onset was estimated from the data. Because we did not model from the time of initial infection, we cannot compute the basic reproductive number R0, which assumes fully susceptible target cell population. Rather, we now clarify that what we compute is the reproductive number at symptom onset, Rs0. Because the number of target cells is likely reduced by the time of symptom onset, Rs0 is below R0 by definition, thus our finding is still reasonable.

The effect of antiviral therapies on viral dynamics was discussed together with other comments below. We added new material on the comparison of our results with those in recent papers on Lines 334-338 on Page17.

2. The predictions of the effects of drugs (Fig 3) show that treatment initiation before the peak in viremia is necessary to curtail overall viral load, whereas treatment initiation post the peak is unlikely to have a major effect, at least with entry and viral production inhibitors. I wonder if this is because of the metric used to assess the effect of the drugs, namely the area under the curve of the viral load (AUC) (line 299). The viral load in the untreated case rises following infection, attains a peak, and then declines naturally. The change in viral load in the process is over many orders of magnitude, starting from ~100

and reaching ~10^6 or 10^8 copies/ml at the peak (Fig. 1). In such a case, the major contribution to the AUC will come from viral load around the peak. It is no surprise thus that treatments started before the peak suppress this contribution and appear to have an effect, whereas those that start post the peak have already seen the major contribution made. It is thus important to assess whether alternative metrics, which may be more indicative of disease severity, would behave similarly. If the disease can be brought under control quickly even with late treatment initiation, so that a cytokine storm is prevented, it might still be worth the effort. Such aspects are missed in the present analysis. With the one drug class that does show an effect with late initiation, I found the prediction of the viral load declining faster with late initiation (and even crossing over) than with early initiation difficult to understand (Fig. 3C). The authors do not offer an explanation of this behavior, which they should be able to using their model. Thus, I find the inferences of the effects of different drug classes and timing of treatment initiation not satisfactory.

Reply to major comment 2:

It is very important to find metrics of clinical severity of COVID-19. Recently, it was reported that a combined metric of several cytokines is associated with severe pneumonia. We are planning a clinical study to identify such metrics in Japan. However, a severity index or other biomarkers were not consistently reported along with longitudinal viral load data in the studies we analyzed. Therefore, we used the area under the viral load curve (AUC) as a metric, as was done in the Goncalves paper that you quoted above.

We revised our analysis of the effects of drugs that promote cytotoxicity. We found that if such drugs were initiated after the viral load peak (i.e., late initiation), then the drugs effectively enhance viral decay (since fewer infected cells remain to produce virus) and reduce the AUC. Meanwhile, if such drugs were initiated before the viral load peak (i.e., early initiation), the drug with maximum efficacy in this simulation (which doubles the rate of infected cell death) was to delay the time of the viral load peak rather than enhancing the viral load decay, and therefore had relatively mild effects on AUC reduction. This is because drugs promoting cytotoxicity do not block further ongoing *de novo* infection, the antiviral effects depend on target cell availability. See the yellow curve in **Fig 2C**. Theoretically, drugs increasing the infected cell death by 10-fold or more (so

infected cells are removed immediately after infection), a rapid viral load decline would be expected even under early treatment initiation.

【Minor comments】

1. Abstract: What is meant by 'severity' is not clear.

Reply to minor comment 1:

In this revised version, we avoided using the term "severity" as our analysis focused on viral load only. Severity information was not consistently available from the patients in different studies.

Line 99: The authors say that they are not in a position to assess the severity of infection of the SARS-CoV-2 patients studied. Yet, with MERS-CoV data, the distinction between mild and severe is made. It may help to specify the fate of the SARS-CoV-2 patients studied - did they require hospitalization, suffer mortality, etc.?

Reply to minor comment 2:

The same reply for **minor comment 1**. Also, we no longer make a distinction between severe and mild MARS-CoV patients.

2. Line 275: please say what is novel about the model.

Reply to minor comment 3:

We no longer use the word 'novel' to describe our model because the model was previously developed by our group. The novelty is in the framework we used to compare the viral dynamics of different corona viruses.

3. Line 295: It may be cleaner to define the Heaviside function as H(t-t*).

Reply to minor comment 4:

We now describe the function more clearly: "$H(t)$ is a Heaviside step function defined as $H(t) = 0$ if $t < t^*$: otherwise $H(t) = 1$"

4. Line 304: It may help to define what is meant by symptom onset.

Reply to minor comment 5:

The symptom onset is the timing of appearance of any COVID-19 related symptoms (fever, cough, and shortness of breath) reported by patients. We now mention this in the paper.

Reviewer #2 in PNAS MS# 2020-04882:
Suitable Quality?:Yes
Sufficient General Interest?:Yes
Conclusions Justified?:Yes
Clearly Written?:Yes
Procedures Described?:Yes
Supplemental Material Warranted?:Yes

Comments on Significance Statement:
Consider replacing the work "curative" I find it a little imprecise here.

【Comments】
This is a paper modelling the within host viral dynamics of SARS-CoV-2 compared to other coronaviruses, and the projected impact of different antivirals. I think there needs to be clarification of why this model formulation and some of the model fitting decisions were made and how this impacts results. There also could be more use of inter-patient variation in the projected impact of the antiviral. Throughout I would suggest a checking of language to be precise about what exactly is being considered here and what this means in terms of treatment, impact on viral dynamics and infectivity.

Reply to Reviewer summary:

The paper has been updated for better clarifications of models, computational process, and interpretation of the results.

1. Line 33: What does it mean for infection dynamics to be more severe? It would be good to be clear from the outset and throughout the paper that this paper is considering treatment with antivirals targeting viral replication, not the downstream effects of virus that can lead to observed symptoms. Both could be considered treatments, but only one is an antiviral and that is what is considered here.

Reply to comment 1:

We avoided using the term "severity" since our models only consider viral load (information of clinical severity was not consistently available). In assessment of treatment effect, we focused on the difference in cumulative viral

load between on and off treatment, and not downstream processes such as inflammation.

2. Line 56: A rogue "¥body"

Reply to comment 2:

We removed it.

3. Line 97: Could the p-values be reported as <....? How is mild and severe MERS-CoV defined? Have any of the SARS-CoV-2 cases had severity defined?

Reply to comment 3:

We reported the *p*-value not just the indication whether it is lower or larger than a significance level following the APA guideline (https://apastyle.apa.org/instructional-aids/numbers-statistics-guide.pdf). In the revised version we no longer discuss mild and severe disease.

4. Line 101: "cases infection" feels a bit imprecise here, could you explain more clearly what you mean? We are only looking at infections that have taken off so the currently wording could be a little unclear.

Reply to comment 4:

We modified our terminology throughout the manuscript.

【Conclusion】

I found the first section of the conclusion talking about transmission a little jarring. I think it would be helpful to motivate both treatment and transmission control here. Though it is seemingly obvious that viral load links with probability of transmission- has this actually been shown anywhere? This would be helpful to discuss, and what shape this relationship could be given what is known about other viruses transmitted in similar ways. How would the % reduction in AUC as reported here, translate to infectivity reduction? I'm not expecting answers to this, but I think it is worth discussing that these things needs to be considered for this to be informative about reduction in transmission due to antivirals.

Reply to comments on conclusion:

We have rewritten the discussion with less emphasis on transmission. Now we only say the following: Although the connection between SARS-CoV-2 viral load and transmissibility has yet to be established, the connection has been suggested for the other viruses [20-22] and may soon be identified for SARS-CoV-2 as suggested by recent modelling [23].

【Methods】

1. Line 237: Assuming those under limit of detection (LOD) were at limit of detection seems a little strange, as you could end up with the model trying to fit to some kind of non-zero equilibrium at the LOD if there were repeated measurements there. Could the authors discuss how this could influence the results? I think this also leads to what seems to me to be a weird results for MERS COV ID10 , ID11 and ID15-17 throat swab, where there is a very late viral increase that does not have any data points to support it. How does this influence the results?

Reply to comment 1 on methods:

We agree this assumption is unreasonable given that may data points are given at the detection limit, which generally implies they are below this limit and only plotted this way for convenience. In our current analysis, we handled these points as censored data in the software package we used for fitting, i.e. Monolix – see Methods section of the paper for further details

2. The authors appear to use a target cell limited model. Could they please discuss why they made this choice and how this will influence their results compared to an immune system controlled model? This also needs to be clear from the beginning of the paper that this type of model was used.

Reply to comment 2 on methods:

We now begin the methods section with a justification for using a target cell limited model.

3. A lot of effort has gone in in the fitting to using a model that estimates the variation in parameters. Is this propagated through to the antiviral model? From the figures it does necessarily look like it. If not, why not? This would

seem important to consider as day 2 symptoms may correspond to a range of future viral dynamics and therefore antiviral impact.

Reply to comment 3 on methods:

Thank you for this suggestion. To see if inter-individual variation in parameters would change the conclusions, we studied several different scenarios varying the time of therapy initiation (1 or 4 days from symptom onset, which are before and after the estimated peak viral load in our dataset) under hypothetical drug therapies possessing different antiviral mechanisms with 95% inhibition rate. In the following figure, which we now include as **Fig S7** in the revised paper, we found similar trends as observed in our **Fig. 2ABC** in which we used the median values of our estimated parameters. This is now discussed on Lines 297-299 on Page 14 in the revised manuscript.

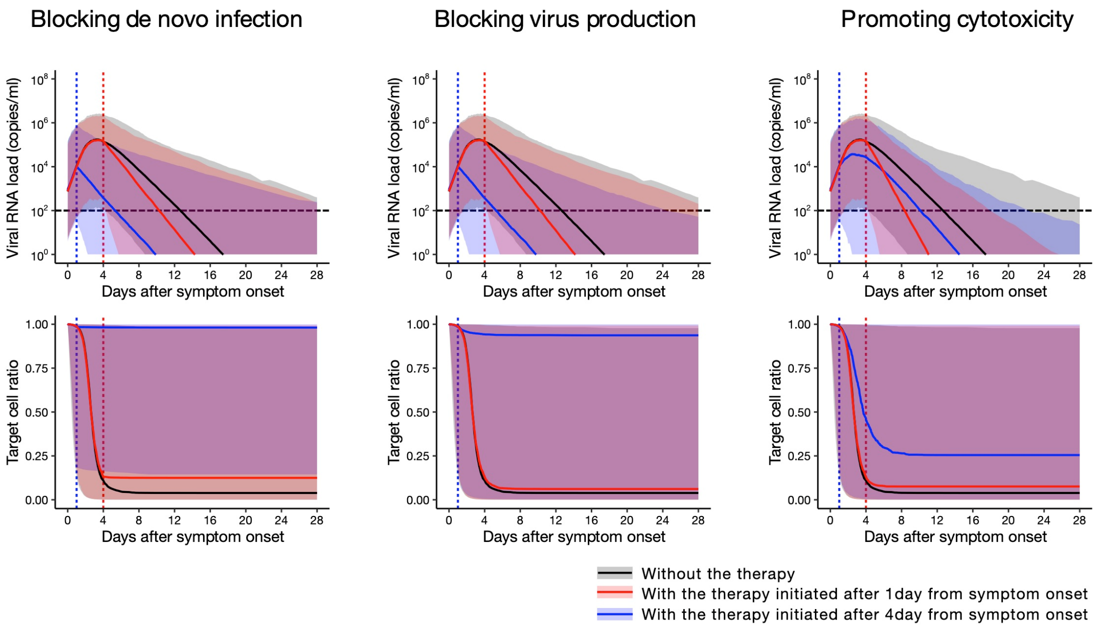

Fig. L1. Predicted outcomes under anti-SARS-CoV-2 monotherapies considering inter-individual variation in parameters. Expected viral load and uninfected target cell proportion trajectories with and without treatment for the three different treatments. The black curves are without treatment. The blue and red curves are with treatment (efficacy is 95%) initiated at 1 days and at 4 days since symptom onset, respectively. The shaded regions correspond to the 95% predictive intervals.

References

1. Zou L, Ruan F, Huang M, Liang L, Huang H, Hong Z, et al. SARS-CoV-2 Viral Load in Upper Respiratory Specimens of Infected Patients. N Engl J Med. 2020;382(12):1177-9. Epub 2020/02/20. doi: 10.1056/NEJMc2001737. PubMed PMID: 32074444; PubMed Central PMCID: PMCPMC7121626.

2. Munster VJ, Feldmann F, Williamson BN, van Doremalen N, Pérez-Pérez L, Schulz J, et al. Respiratory disease in rhesus macaques inoculated with SARS-CoV-2. Nature. 2020. Epub 2020/05/13. doi: 10.1038/s41586-020-2324-7. PubMed PMID: 32396922.

3. Kim ES, Chin BS, Kang CK, Kim NJ, Kang YM, Choi JP, et al. Clinical Course and Outcomes of Patients with Severe Acute Respiratory Syndrome Coronavirus 2 Infection: a Preliminary Report of the First 28 Patients from the Korean Cohort Study on COVID-19. Journal of Korean medical science. 2020;35(13):e142. Epub 2020/04/04. doi: 10.3346/jkms.2020.35.e142. PubMed PMID: 32242348; PubMed Central PMCID: PMCPMC7131901.

4. Lescure FX, Bouadma L, Nguyen D, Parisey M, Wicky PH, Behillil S, et al. Clinical and virological data of the first cases of COVID-19 in Europe: a case series. The Lancet Infectious diseases. 2020;20(6):697-706. Epub 2020/04/01. doi: 10.1016/s1473-3099(20)30200-0. PubMed PMID: 32224310; PubMed Central PMCID: PMCPMC7156120.

5. Wölfel R, Corman VM, Guggemos W, Seilmaier M, Zange S, Müller MA, et al. Virological assessment of hospitalized patients with COVID-2019. Nature. 2020;581(7809):465-9. Epub 2020/04/03. doi: 10.1038/s41586-020-2196-x. PubMed PMID: 32235945.

6. Young BE, Ong SWX, Kalimuddin S, Low JG, Tan SY, Loh J, et al. Epidemiologic Features and Clinical Course of Patients Infected With SARS-CoV-2 in Singapore. Jama. 2020;323(15):1488-94. Epub 2020/03/04. doi: 10.1001/jama.2020.3204. PubMed PMID: 32125362; PubMed Central PMCID: PMCPMC7054855 Sanofi and Roche. Dr Wang reported receiving grants from the Ministry of Health, Singapore. No other disclosures were reported.

7. Al-Abdely HM, Midgley CM, Alkhamis AM, Abedi GR, Lu X, Binder AM, et al. Middle East Respiratory Syndrome Coronavirus Infection Dynamics and Antibody Responses among Clinically Diverse Patients, Saudi Arabia. Emerging infectious diseases. 2019;25(4):753-66. Epub 2019/03/19. doi: 10.3201/eid2504.181595. PubMed PMID: 30882305; PubMed Central PMCID: PMCPMC6433025.

8. Oh MD, Park WB, Choe PG, Choi SJ, Kim JI, Chae J, et al. Viral Load Kinetics of MERS Coronavirus Infection. N Engl J Med. 2016;375(13):1303-5. Epub 2016/09/30. doi: 10.1056/NEJMc1511695. PubMed PMID: 27682053.

9. Peiris JS, Chu CM, Cheng VC, Chan KS, Hung IF, Poon LL, et al. Clinical progression and viral load in a community outbreak of coronavirus-associated SARS pneumonia: a prospective study. Lancet (London, England). 2003;361(9371):1767-72. Epub 2003/06/05. doi: 10.1016/s0140-6736(03)13412-5. PubMed PMID: 12781535; PubMed Central PMCID: PMCPMC7112410.

10. Perelson AS, Essunger P, Cao Y, Vesanen M, Hurley A, Saksela K, et al. Decay characteristics of HIV-1-infected compartments during combination therapy. Nature. 1997;387(6629):188-91. Epub 1997/05/08. doi: 10.1038/387188a0. PubMed PMID: 9144290.

11. Perelson AS, Neumann AU, Markowitz M, Leonard JM, Ho DD. HIV-1 dynamics in vivo: virion clearance rate, infected cell life-span, and viral generation time. Science. 1996;271(5255):1582-6. Epub 1996/03/15. PubMed PMID: 8599114.

12. Neumann AU, Lam NP, Dahari H, Gretch DR, Wiley TE, Layden TJ, et al. Hepatitis C viral dynamics in vivo and the antiviral efficacy of interferon-alpha therapy. Science. 1998;282(5386):103-7. Epub 1998/10/02. PubMed PMID: 9756471.

13. Baccam P, Beauchemin C, Macken CA, Hayden FG, Perelson AS. Kinetics of influenza A virus infection in humans. J Virol. 2006;80(15):7590-9. Epub 2006/07/15. doi: 80/15/7590 [pii]
10.1128/JVI.01623-05. PubMed PMID: 16840338; PubMed Central PMCID: PMC1563736.

14. Banerjee S, Guedj J, Ribeiro RM, Moses M, Perelson AS. Estimating biologically relevant parameters under uncertainty for experimental within-host murine West Nile virus infection. J R Soc Interface. 2016;13(117). doi: 10.1098/rsif.2016.0130. PubMed PMID: 27075003; PubMed Central PMCID: PMCPMC4874436.

15. Best K, Guedj J, Madelain V, de Lamballerie X, Lim SY, Osuna CE, et al. Zika plasma viral dynamics in nonhuman primates provides insights into early infection and antiviral strategies. Proc Natl Acad Sci U S A. 2017;114(33):8847-52. Epub 2017/08/03. doi: 10.1073/pnas.1704011114. PubMed PMID: 28765371; PubMed Central PMCID: PMCPMC5565429.

16. Gonçalves A, Bertrand J, Ke R, Comets E, de Lamballerie X, Malvy D, et

al. Timing of antiviral treatment initiation is critical to reduce SARS-CoV-2 viral load. CPT Pharmacometrics Syst Pharmacol. 2020. Epub 2020/06/20. doi: 10.1002/psp4.12543. PubMed PMID: 32558354.

17. Dobrovolny HM. Quantifying the effect of remdesivir in rhesus macaques infected with SARS-CoV-2. Virology. 2020;550:61-9. doi: 10.1016/j.virol.2020.07.015. PubMed PMID: 32882638; PubMed Central PMCID: PMCPMC7443325.

18. Hernandez-Vargas EA, Velasco-Hernandez JX. In-host Mathematical Modelling of COVID-19 in Humans. Annu Rev Control. 2020. doi: 10.1016/j.arcontrol.2020.09.006. PubMed PMID: 33020692; PubMed Central PMCID: PMCPMC7526677.

19. Goyal A, Cardozo-Ojeda EF, Schiffer JT. Potency and timing of antiviral therapy as determinants of duration of SARS CoV-2 shedding and intensity of inflammatory response. medRxiv. 2020:2020.04.10.20061325. doi: 10.1101/2020.04.10.20061325.

20. Fraser C, Hollingsworth TD, Chapman R, de Wolf F, Hanage WP. Variation in HIV-1 set-point viral load: epidemiological analysis and an evolutionary hypothesis. Proc Natl Acad Sci U S A. 2007;104(44):17441-6. Epub 2007/10/24. doi: 10.1073/pnas.0708559104. PubMed PMID: 17954909; PubMed Central PMCID: PMCPMC2077275.

21. Major M, Gutfraind A, Shekhtman L, Cui Q, Kachko A, Cotler SJ, et al. Modeling of patient virus titers suggests that availability of a vaccine could reduce hepatitis C virus transmission among injecting drug users. Sci Transl Med. 2018;10(449). Epub 2018/07/13. doi: 10.1126/scitranslmed.aao4496. PubMed PMID: 29997251; PubMed Central PMCID: PMCPMC6552668.

22. Yamin D, Gertler S, Ndeffo-Mbah ML, Skrip LA, Fallah M, Nyenswah TG, et al. Effect of Ebola progression on transmission and control in Liberia. Ann Intern Med. 2015;162(1):11-7. Epub 2014/10/28. doi: 10.7326/m14-2255. PubMed PMID: 25347321; PubMed Central PMCID: PMCPMC4402942.

23. Ke R, Zitzmann C, Ribeiro RM, Perelson AS. Kinetics of SARS-CoV-2 infection in the human upper and lower respiratory tracts and their relationship with infectiousness. medRxiv. 2020:2020.09.25.20201772. doi: 10.1101/2020.09.25.20201772.
