# Supplementary material for: A quantitative model used to compare within-host SARS-CoV-2, MERS-CoV, and SARS-CoV dynamics provides insights into the pathogenesis and treatment of SARS-CoV-2
Source: PLoS Biol. 2021 Mar 22;19(3):e3001128. doi: 10.1371/journal.pbio.3001128 (PMC7984623; doi:10.1371/journal.pbio.3001128)
Supplement: Attachment — Submitted filename: PBP_final.docx [file pbio.3001128.s023.docx]

**Response to reviewer comments**

Kwang Su Kim, Keisuke Ejima, Shoya Iwanami, Yasuhisa Fujita, Hirofumi Ohashi, Yoshiki Koizumi, Yusuke Asai, Shinji Nakaoka, Koichi Watashi, Kazuyuki Aihara, Robin N. Thompson, Ruian Ke, Alan S. Perelson

and Shingo Iwami

February 1, 2021

We would like to thank the reviewers for their thorough reading of the manuscript and their excellent comments and suggestions. Below we present the reviewers’ comments in black and provide our responses in red. All the changes made in response to the reviewers’ comments in PBIOLOGY-D-20-03579R1 have been addressed in the revised version of the manuscript. We believe that the manuscript has been considerably improved by these changes. All coauthors agreed with these changes.

**Requests from the editors:**

Thank you for submitting your revised Research Article entitled "Quantitative comparison of SARS-CoV-2, MERS-CoV and SARS-CoV dynamics: Implications for therapy" for publication in PLOS Biology. I've now obtained advice from two of the original reviewers and have discussed their comments with the Academic Editor. Please note that reviewer #3 is the person who had told us that they had reviewed your study for another journal, but to whose comments you responded. Reviewer #2 is the original PLOS Biology reviewer #2. Reviewer #1 was not able to re-review.

Based on the reviews, we will probably accept this manuscript for publication, assuming that you will modify the manuscript to address the remaining points raised by the reviewers. Please also make sure to address the data and other policy-related requests noted at the end of this email.

a) Please attend to the remaining requests from reviewer #3.

**Reply to Request a):**

We addressed all the reviewers’ comments (See Page 4).

b) Please update your co-author's funding information (as discussed in our email conversation).

**Reply to Request b):**

We updated the funding information accordingly.

c) Please change your title to something that makes it clear that this is a modelling study, and that reduces the emphasis on implications for therapy. We suggest the following: "A quantitative model to compare within-host SARS-CoV-2, MERS-CoV and SARS-CoV dynamics provides insights into the pathogenesis of SARS-CoV-2"

**Reply to Request c):**

We changed the title to “A quantitative model used to compare within-host SARS-CoV-2, MERS-CoV and SARS-CoV dynamics provides insights into the pathogenesis and treatment of SARS-CoV-2”. We trust this is acceptable.

d) Please address my Data Policy requests (see further down) by supplying the data and/or code that underlies the Figures.

**Reply to Request d):**

We supplied the data that underlies the Figures as follows:

1. **S1 Data**. The data to reproduce **Fig 1**.

2. **S2 Data**. The data to reproduce **Fig 2**.

3. **S3 Data**. The data to reproduce **Fig 3**.

4. **S4 Data**. The data to reproduce **Fig S1**.

5. **S5 Data**. The data to reproduce **Fig S2**.

6. **S6 Data**. The data to reproduce **Fig S3**.

7. **S7 Data**. The data to reproduce **Fig S4**.

8. **S8 Data**. The data to reproduce **Fig S5**.

9. **S9 Data**. The data to reproduce **Fig S6**.

10. **S10 Data**. The data to reproduce **Fig S7**.

**Reviewer #3 :**

I thank the authors for their thorough responses to my previous comments. I think the manuscript is more focused and more clearly states what the modelling can show.

**Reply to Reviewer summary:**

We appreciate your help in improving the quality of our manuscript.

I just have two remaining comments:

【Comments】

1. For the target cell models it is mentioned that the parameters implicitly include the immunity. Do these parameters (delta for example as mentioned in the discussion) change over time. If not, they are missing an important thing that immunity does to control infection which is proliferate over time. Could the authors please comment on this.

**Reply to major comment 1:**

We assumed the death rate of infected cells, delta, is constant. However, delta can be made time-dependent as was done by Stafford et al. [1] in the case of an HIV model with extensive viral load data, or equations can be introduced to explicitly model effector cell responses [2, 3]. These approaches could be reflected in extended versions of our model if relevant data and supporting evidence becomes available. We mentioned this point as a limitation and a future direction of our study (See Page 17).

2. I would still like to see a little more discussion in line 306-313 on the endpoints used in trials that are generally not viral, and how what it is that is important for the drugs to be able to do (I would think reduce severity and death) and how this is not what is discussed in this paper.

**Reply to major comment 2:**

Based on your suggestion we expanded our discussion of clinical endpoints (See Page 16).

**Reference**

1.	Stafford MA, Corey L, Cao Y, Daar ES, Ho DD, Perelson AS. Modeling plasma virus concentration during primary HIV infection. J Theor Biol. 2000;203(3):285-301. Epub 2000/03/16. doi: 10.1006/jtbi.2000.1076. PubMed PMID: 10716909.

2.	Baral S, Antia R, Dixit NM. A dynamical motif comprising the interactions between antigens and CD8 T cells may underlie the outcomes of viral infections. Proc Natl Acad Sci U S A. 2019;116(35):17393-8. Epub 2019/08/16. doi: 10.1073/pnas.1902178116. PubMed PMID: 31413198; PubMed Central PMCID: PMCPMC6717250.

3.	Conway JM, Perelson AS. Post-treatment control of HIV infection. Proc Natl Acad Sci U S A. 2015;112(17):5467-72. Epub 2015/04/15. doi: 10.1073/pnas.1419162112. PubMed PMID: 25870266; PubMed Central PMCID: PMCPMC4418889.
